# Quorum Sensing System of *Ruegeria mobilis* Rm01 Controls Lipase and Biofilm Formation

**DOI:** 10.3389/fmicb.2018.03304

**Published:** 2019-01-09

**Authors:** Ying Su, Kaihao Tang, Jiwen Liu, Yan Wang, Yanfen Zheng, Xiao-Hua Zhang

**Affiliations:** ^1^College of Marine Life Science, Ocean University of China, Qingdao, China; ^2^Weifang Engineering Vocational College, Weifang, China; ^3^Laboratory for Marine Ecology and Environmental Science, Qingdao National Laboratory for Marine Science and Technology, Qingdao, China

**Keywords:** marine particles, quorum sensing, quorum quenching, *Ruegeria mobilis*, biofilm formation, extracellular enzyme

## Abstract

Quorum sensing (QS) promotes *in situ* extracellular enzyme (EE) activity via the exogenous signal *N*-acylhomoserine lactone (AHL), which facilitates marine particle degradation, but the species that engage in this regulatory mechanism remain unclear. Here, we obtained AHL-producing and AHL-degrading strains from marine particles. The strain *Ruegeria mobilis* Rm01 of the *Roseobacter* group (RBG), which was capable of both AHL producing and degrading, was chosen to represent these strains. We demonstrated that Rm01 possessed a complex QS network comprising AHL-based QS and quorum quenching (QQ) systems and autoinducer-2 (AI-2) perception system. Rm01 was able to respond to multiple exogenous QS signals through the QS network. By applying self-generated AHLs and non-self-generated AHLs and AI-2 QS signal molecules, we modulated biofilm formation and lipase production in Rm01, which reflected the coordination of bacterial metabolism with that of other species via eavesdropping on exogenous QS signals. These results suggest that *R. mobilis* might be one of the participators that could regulate EE activities by responding to QS signals in marine particles.

## Introduction

Marine particles are typically derived from organic polymers, phytoplankton, bacteria, fecal pellets, mineral materials, and other suspended matter characteristic of a given water mass (Silver and Alldredge, 1981). They are reservoir for numerous elements and compounds, such as trace metals, C, N, protein, carbohydrate and lipid (Alldredge, 1979), and are responsible for the delivery of the surface organic matter to the seafloor (Alldredge and Silver, 1988). During the sinking process, large numbers of heterotrophic bacteria continuously approach, and colonize these particles (Kiørboe et al., 2002). The heterotrophic bacteria secrete various extracellular enzymes (EEs) to hydrolyze macromolecules into small molecules that are readily absorbed into cells (Cho and Azam, 1988), which promotes marine particle decomposition (Smith et al., 1992; Azam and Long, 2001). Moreover, bacterial abundance and cell-specific EE activity are much higher in marine particles than in ambient seawater (Smith et al., 1992), which indicates the activation of EE production in marine particles. However, studies investigating the regulatory mechanisms of bacterial EE production in marine particles are limited.

Quorum sensing (QS) is the regulation of gene expression in response to high bacterial density. In the QS regulatory process, QS autoinducers (AIs) are key factors delivering messages among bacterial communities, i.e., *N*-acylhomoserine lactones (AHLs) in Gram-negative bacteria (Williams, 2007; Case et al., 2008), autoinducing peptides (AIPs) in Gram-positive bacteria (Lyon and Novick, 2004) and autoinducer-2 (AI-2) in both Gram-positive and Gram-negative bacteria (Bassler and Losick, 2006). Amendments of QS signal AHLs and AI-2 affected EE production in marine particles (Hmelo et al., 2011; Krupke et al., 2016) and in *Trichodesmium* consortia (Van Mooy et al., 2012), which demonstrated the regulatory roles of QS on element cycling in oceans. Disruption of QS pathway is another gene regulatory mechanism, termed as quorum quenching (QQ). QQ could be achieved by species producing inhibition molecules (Manefield et al., 2002) or AHL-degrading enzymes AHL lactonases and AHL acylases (Dong and Zhang, 2005). In marine ecosystem, enzymatic QQ activities have been observed in seawater (Hmelo and Van Mooy, 2009) and *Trichodesmium* colonies (Van Mooy et al., 2012). Although the QS and QQ bacteria are prevalent in marine particles (Gram et al., 2002; Romero et al., 2011), whether they are responsible for the QS-regulated EE production in marine particles is still unclear.

Species belonging to *Roseobacter* group (RBG) are widespread (Wagner-Dobler and Biebl, 2006; Wietz et al., 2010) and prefer a surface-attached lifestyle (Gram et al., 2002; Zan et al., 2014). They are main forces for AHL producing activities in marine particles (Gram et al., 2002; Doberva et al., 2015). According to a genome analysis, QS in RBG is conserved and widespread thus might participate in multiple metabolisms controlling (Cude and Buchan, 2013). As revealed by several RBG species, QS regulate phenotypic traits related to ecological success especially in particle-attached lifestyle, e.g., flagellar motility and biofilm formation in *Ruegeria* sp. KLH11 (Zan et al., 2012), antimicrobial indigoidine biosynthesis in *Phaeobacter* sp. strain Y4I (Cude et al., 2015) and tropodithietic acid (TDA) production in *Phaeobacter inhibens* DSM 17395 (Berger et al., 2011). *Ruegeria mobilis*, another common species of the RBG, is capable of biofilm formation and TDA production as well (Porsby et al., 2008; D’Alvise et al., 2014), which may allow them to invade already formed bacterial biofilms and occupy advantageous status on marine particles (Rao et al., 2005). Although the QS circuit in *R. mobilis* has not been identified, two putative AHL synthases differing from the reported AHL synthases were proposed (Sonnenschein et al., 2016) and the potential role of QS on biofilm formation was suggested by controlling the intracellular signal compound cyclic dimeric guanosinmonophosphate (c-di-GMP) concentration (Srivastava and Waters, 2012; D’Alvise et al., 2014).

In this study, we aimed to extend investigation of QS-regulated marine particle degradation. We collected marine particle samples from the Yellow Sea of China and reported (i) regulations of EE activities in marine particles with exogenous AHL; (ii) the QS and QQ strains isolated from marine particles; (iii) QS networks of a RBG species, *R. mobilis* Rm01; and (iv) the controls of biofilm formation and EE production in Rm01 by diverse QS signals. This study represents the comprehensive analysis of bacterial assemblage of QS and QQ strains in marine particles; indicates the complex interspecies signaling conducted by Rm01 and provides perspective in revealing mechanisms of QS-regulated marine particle degradation.

## Materials and Methods

### Marine Particle Collection

The marine particle samples were collected in the Yellow Sea of China on board R/V “Dong Fang Hong 2” in October 2015. Approximately 75 L of seawater at the depth of 2–10 m was obtained via an injection pump at six stations (Supplementary Figure 1). The *in situ* seawater was filtered through 3-μm and 0.22-μm GTTP Isopore membrane filters (Millipore, Ireland) in succession immediately after sample collection. The marine particles collected on 3-μm filters were rinsed and re-suspended with 10 ml of 0.22-μm-filtered *in situ* seawater and transferred into a sterile 50-ml polypropylene (PP) tube for temporal storage.

### Regulations of EE Activity in Marine Particles by Exogenous AHL

An aliquot of 500 μl of condensed marine particle sample in H33 station was pipetted into a sterile 50-ml PP tube, and then the volume was expanded to 10 ml incubation systems with 0.22-μm-filtered *in situ* seawater. The marine particle incubations were divided into three treatment groups amended with exogenous *N*-(3-oxo-octanoyl)-L-homoserine lactone (3OC8-HSL) (a serial concentrations of 50, 100, 500, and 1000 nM), 3OC8-HSL solvent DMSO (negative control) and 0.22-μm-filtered *in situ* seawater (blank control), respectively. Each treatment included triplicate incubations maintained at 25°C (approximately equivalent to the surface seawater temperature). After incubation of 6 and 24 h, an aliquot of 200 μl sample was centrifuged at 12,000 rpm for 5 min to collect supernatant used for EE assays. Following the methods described by Hoppe and Hmelo (Hoppe, 1993; Hmelo et al., 2011), ten EE activity were assayed using fluorescent substrates, i.e., MUF-α-glucopyranoside (for α-glucosidase activity), MUF-β-glucopyranoside (for β-glucosidase activity), MUF-β-D-xylopyranoside (for β-xylosidase activity), MUF-α-D-mannopyranoside (for mannosidase activity), MUF-β-D-cellobioside (for cellulase activity), MUF*-N*-acetyl-β-D-galactosaminide (for galactosaminidase activity), MUF-*N*-acetyl-β-D-glucosamine (for chitobiase activity), MCA-leucine (for aminopeptidase activity), MUF-phosphate (for phosphatase activity) and MUF-butyrate (for lipase activity) (all fluorescent substrates were purchased from Sigma-Aldrich). Released fluorescent signals were detected with a Fluoroskan Ascent FL multi-well plate reader (Thermo) with continuous reading at 2-min intervals during the enzyme activity assay over 1 h. The variations of EE activities were calculated by subtracting the EE activities of blank controls from that of the corresponding treatments. The excitation and emission characteristics of the fluorophores were previously programmed into the instrument (364 and 445 nm, respectively, for MUF; 380 and 440 nm, respectively, for MCA). Standard curves were constructed using the standard fluorophores MUF and MCA (Sigma-Aldrich).

### Isolation and Identification of Bacterial Strains From Marine Particles

The condensed particle sample was serially diluted from 10^-1^ to 10^-6^ with 0.22-μm-filtered *in situ* seawater and 100 μl of each dilution was spread on 2216E marine agar (MA, Difco) plates. Colonies appeared were transferred three to four times on MA plates to obtain pure isolates. Further identification of the isolates relied on PCR using the B8F (5′-AGAGTTTGATCCTGGCTCAG-3′) and B1510 (5′-GGTTACCTTGTTACGACTT-3′) primers (Weisburg et al., 1991) for 16S rRNA gene amplification followed by sequencing at BGI (Qingdao, China). Sequence similarities between isolates and their closest relatives were calculated using the EzTaxon-e server (Kim et al., 2012).

### Screening for AHL-Producing and AHL-Degrading Bacterial Species

Screening for AHL-producing and AHL-degrading species was conducted using the reporter strain *Agrobacterium tumefaciens* (pCF218) (pCF372) A136 (Zhu et al., 2003). AHL-producing activity was confirmed by the cross-feeding method according to Chu et al. (2011). In brief, the reporter strain A136 and the tested strains were streaked adjacently on the same agar plates enriched with modified marine broth medium (MB 2216E; Difco, Detroit, MI, United States) and 0.5% X-Gal. The pH of MB medium was adjusted to 6.7 in avoid of the spontaneous alkaline hydrolysis of AHLs (Decho et al., 2009). After co-incubation at 28°C for 24 h, positive results were confirmed by the presence of indigo spots on A136.

AHL-degrading activity was detected using the high-throughput method described by Tang (Tang et al., 2013). *N*-hexanoyl-L-homoserine lactone (C6-HSL, representative for AHLs with short acyl chains) and *N*-dodecanoyl-L-homoserine lactone (C12-HSL, representative for AHLs with long acyl chains) were used as the substrates. Briefly, C6-HSL or C12-HSL was mixed with the bacterial culture and MB medium (negative control) and maintained at 28°C for 24 h. After incubation, the supernatant was obtained, mixed with an A136 X-gal assay solution (an overnight broth culture of A136 inoculated in AT minimal glucose medium and mixed with 0.5% X-Gal) in 96-well plates and incubated at 28°C for another 24 h. Positive results were indicated by reduced indigo color compared to the negative control.

### Identification of AHLs Produced and Degraded by *Ruegeria mobilis* Rm01

A RBG species *R. mobilis* Rm01 was identified for holding both AHL-producing and AHL-degrading abilities. The initial time for QS and QQ activities were primarily detected for further identification of AHLs produced and degraded by Rm01.

AHLs secreted by Rm01 were extracted using an ethyl acetate protocol (Shaw et al., 1997; Zhu et al., 1998) and further evaluated by gas chromatography-mass spectrometer (GC-MS) system (Wagner-Dobler et al., 2005; Cataldi et al., 2007). Briefly, the GC-MS analysis was performed using a GC system 6890 N connected to an Agilent-5973 mass selective detector (Agilent Technologies, United States). One microliter of sample was injected into a HP-5 MS capillary column (30 m × 0.25 mm ID and 0.25 μm film thickness) and analyzed in split mode. Helium (99.99%) was used as a carrier gas at a flow rate of 0.8 ml min^-1^. The oven temperature was held at 150°C for 3 min and then increased to 280°C at a rate of 15°C min^-1^. The mass spectrometer was run in single ion monitoring (SIM) mode at m/z 143. A compassion of mass spectra and retention time of chromatographic peak with AHL standards (at the final concentration of 1 mg/ml) allowed a quick detection of AHLs in sample.

The AHL degrading ability of Rm01 was detected following the procedures described above with a few modifications. The strain Rm01 was inoculated in 2216E liquid and cultured for 8 h on a shaker (170 rpm) at 28°C. The cells were harvested after centrifugation for 10 min at 4°C and 6,000 rpm and re-suspended in HEPES buffer (20 mM Na-HEPES with 0.5 M NaCl, 10% glycerol, and 0.1% Triton X-100, pH 8.5) for sonication. Additional centrifugation for the lysed cells was conducted at 4°C and 12,000 rpm to obtain the crude enzyme supernatant. The supernatant inactivated by proteinase K (final concentration, 200 μg/ml) was used as the negative control. After co-incubation, the QQ activity of Rm01 was indicated with well-diffusion assays (Gram et al., 2002) using the reporter strains A136 and *Chromobacterium violaceum* VIR24 (Someya et al., 2009). Another acidification test to determine QQ enzymatic mechanisms was conducted according to Yates et al. (2002).

### Identification of QS and QQ Enzyme Encoding Genes in Rm01

Rm01 genomic DNA was obtained using the phenol-chloroform method (Murray and Thompson, 1980) and the whole-genome sequencing was performed using Illumina Hiseq 2000 platform and Pacific Biosciences (PacBio) sequencing at BGI. PacBio sequence read data were assembled *de novo* using Canu version 1.0 (Berlin et al., 2015) and further polished using Illumina sequencing data to resolve single nucleotide errors. The assembled genome sequences were submitted into RAST server for genome annotation (Aziz et al., 2008).

Putative QS (AHL synthase) and QQ enzyme (AHL lactonase or AHL acylase) encoding genes were searched from Rm01 genome through local BLASTP against known QS and QQ enzyme databases. The putative QS and QQ enzyme encoding genes were amplified with primers (Supplementary Table S1) and merged into vector pET-24a. The recombined plasmids were transformed into *Escherichia coli* BL21 (DE3).

The putative QS and QQ genes were heterogeneously expressed in BL21 (DE3) and examined for QS and QQ activities. The recombinant strains were grown to OD_600_ 0.4–0.6 and induced with 0.1 mM IPTG for 12 h on a shaker (150 rpm) at 16°C. AHLs synthesized by QS enzymes expressed in *E. coli* (DE3) were extracted and analyzed by the GC-MS approach. The activity of QQ enzyme expressed in *E. coli* (DE3) was detected by well-diffusion bioassays described above.

### The Influence of AIs on Biofilm Formation and EE Production in Rm01

The influence of diverse AIs on physiological metabolisms in Rm01 was investigated by growing bacterium in MB medium supplemented with 10 μM self-generated *N*-(3-oxo-decanoyl)-L-homoserine lactone (3OC10-HSL), *N*-decanoyl-L-homoserine lactone (C10-HSL) and C12-HSL; non-self-generated 3OC8-HSL, *N*-tetradecanoyl-L-homoserine lactone (C14-HSL) and AI-2; and purified AHL lactonase MomL (0.5 U ml^-1^) (Tang et al., 2015). The reagent solvent DMSO, 1 M PIPES and solution buffer (HEPES buffer supplemented with 25% glycerol) were used as negative controls for AHLs, AI-2 and MomL, respectively. Each treatment included triplicate repeats, and all experiments were repeated at least 3 times.

For biofilm mass quantification, Rm01 was grown in 200 μl of MB medium using 96-well microtiter plate and was maintained at 28°C for 8 h. Biofilm mass quantification was carried out as described previously (Sheikh et al., 2001) with a few modifications. Briefly, the attached cells were stained with 0.2% crystal violet (CV) and washed three times with ddH_2_O. The remaining CV was re-dissolved in 75% ethanol, and finally, the absorbance at 570 nm was monitored with a Tecan Sunrise^TM^ microplate absorbance reader.

Primarily identification of EE production in Rm01 was conducted using fluorescent substrates mentioned above and only lipase was detected in the sterile filtered culture supernatant. For lipase activity assay, Rm01 was grown in 100 ml of MB medium using 500 ml schott flasks and was incubated at 28°C for 24 h. Growth was measured at OD_600_ and sterile culture supernatants were obtained for lipase activity using fluorescent substrates MUF-butyrate. Additionally, the enzyme activities of exogenous AHLs, AI-2 and MomL were also assayed under the same incubation conditions and subtracted from the corresponding assayed lipase activities.

## Results

### Exogenous AHL Regulates EE Production in Marine Particles

**Figure 1:**
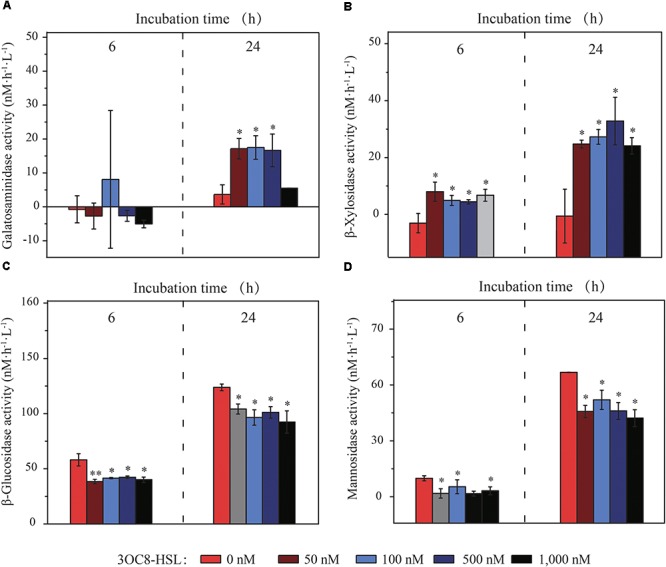
*In situ* galactosaminidase **(A)**, β-xylosidase **(B)**, β-glucosidase **(C)**, and mannosidase **(D)** activities in marine particles treated with 3OC8-HSL (the final concentrations of 3OC8-HSL are shown in different colors). The data are shown as the mean ± standard deviation (SD). The difference between the 3OC8-HSL-treated groups and the un-treated control groups was calculated by Student’s *t*-test (^∗∗^*P* < 0.01; ^∗^*P* < 0.05).

Since GC-MS analyses of AHLs cannot be conducted on R/V “Dong Fang Hong 2”, the AHL 3OC8-HSL, which was previously identified in marine particles (Hmelo et al., 2011), was applied as the exogenous AHL. After incubation for 6 and 24 h, the activity of ten EEs (seven carbohydrases, one aminopeptidase, one lipase and one phosphatase) was assayed. Of the EEs, four carbohydrases were modulated with the addition of 3OC8-HSL (Figure 1). Galactosaminidase activity was up to 3–4-fold higher than that of the control after 24 h, and the activity of initially undetectable β-xylosidase was elevated at both time points. By contrast, β-glucosidase and mannosidase activities were inhibited, and this effect was sustained from 6 to 24 h. The addition of 3OC8-HSL in higher concentrations (100, 500, and 1000 nM) did not enhance the effects induced by 3OC8-HSL in low concentration (50 nM) (Figure 1), which is suggestive of the inducing threshold of 3OC8-HSL. No significant variations were observed in the activities of chitobiase, aminopeptidase, lipase and phosphatase (Supplementary Figure 2). We propose that the modulations on EE activities might be achieved by species possessing QS networks in marine particles.

### AHL-Producing and AHL-Degrading Bacteria in Marine Particles

**Figure 2:**
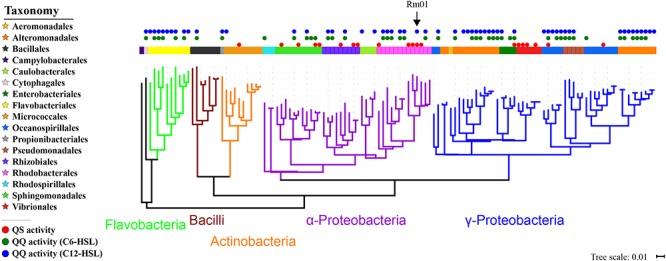
Phylogenetic tree of cultivable bacterial community in marine particles based on the 16S rRNA gene. The arrow indicates the phylogenetic position of *Ruegeria mobilis* Rm01.

In order to figure out the species participating in the QS-regulated EE production, we screened for AHL-producing and AHL-degrading strains from marine particles using the reporter strain A136. A total of 122 bacterial species were identified according to 16S rRNA gene sequence analyses and phylogenetically classified into four phyla: Proteobacteria, Bacteroidetes, Actinobacteria, and Firmicutes. Among these species, 20 and 62 species possessed AHL-producing and AHL-degrading abilities, respectively (Table 1 and Supplementary Figure 3). Most AHL-producing isolates belonged to the orders *Rhodobacterales* (5 species), *Sphingomonadales* (4 species), *Vibrionales* (4 species) and *Rhizobiales* (3 species), while AHL-degrading isolates belonged to the orders *Alteromonadales* (19 species), *Flavobacteriales* (9 species), *Rhodobacterales* (7 species) and *Oceanospirillales* (7 species) (Figure 2). The results demonstrated high proportions of species possessing QS or QQ activities and suggested a large scale of bacteria possibly involving in QS-regulated EE production in marine particles. Notably, the isolate *R. mobilis* Rm01 was found possessing both AHL-producing and AHL-degrading abilities thus it was chosen as representative for further investigations.

**Table 1:** Summary of AHL-producing and/or AHL-degrading bacterial isolates.

Phylum or group	Strains	Identification by EZBioCloud alignment	% identity to EZBioCloud sequence	AHL-producing ability ( 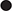 )	AHL-degrading ability ( 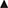 )
*Actinobacteria*	H19-20	*Kocuria palustris* DSM 11925^T^	100	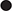	
	H10-56	*Microbacterium aquimaris* JS54-2^T^	98.1		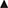
	H19-37	*Nocardioides marinus* CL-DD14^T^	98.7		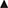
*Cytophagia*	H18-59	*Algoriphagus boritolerans* T-22^T^	98		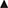
	H01-35	*Croceitalea litorea* CBA3205^T^	96.4		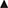
*Flavobacteria*	H33-3	*Dokdonia genika* Cos-13^T^	99.8		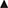
	H33-47	*Empedobacter falsenii* NF 993^T^	98.3		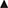
	H33-97	*Maribacter dokdonensis* DSW-8^T^	99.9		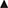
	H33-75	*Meridianimaribacter vietnamensis* KMM 6217^T^	99.5		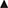
	H19-32	*Muricauda ruestringensis* DSM 13258^T^	97.2		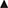
	H33-69	*Non-labens tegetincola* UST030701-324^T^	100		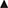
	H19-18	*Tenacibaculum mesophilum* MBIC1140^T^	99.1		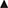
	H01-25	*Tenacibaculum xiamenense* WJ-1^T^	97.6		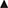
*Alphaproteobacteria*	H33-92	*Aliiroseovarius pelagivivens* GYSW-22^T^	97.3	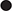	
	H10-30	*Brevundimonas abyssalis* TAR-001^T^	100		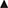
	H19-55	*Brevundimonas nasdae* GTC 1043^T^	99.2		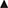
	H10-60	*Citromicrobium bathyomarinum* JF-1^T^	99.8		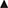
	H33-63	*Erythrobacter citreus* RE35F/1^T^	99.4	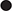	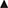
	H18-45	*Erythrobacter gaetbuli* SW-161^T^	98.9		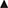
	H19-12	*Erythrobacter pelagi* UST081027-248^T^	100	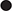	
	H19-15	*Henriciella aquimarina* P38^T^	98		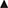
	H33-86	*Henriciella marina* DSM 19595^T^	98.3		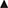
	H19-22	*Hoeflea suaedae* YC6898^T^	98.9		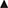
	H33-59	*Hyphomonas atlantica*22II1-22F38^T^	100		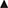
	H19-9	*Jiella aquimaris* LZB041^T^	100	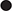	
	H10-43	*Labrenzia aggregata* IAM 12614^T^	100		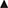
	H10-55	*Labrenzia alba* CECT 5094^T^	98.7		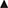
	H19-11	*Mesorhizobium tamadayense* Ala-3^T^	97.4	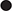	
	H19-35	*Mesorhizobium thiogangeticum* SJT^T^	97.4	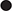	
	H33-8	*Nautella italica* CCUG 55857^T^	97.8	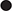	
	H33-70-1	*Oceanicaulis stylophorae* GISW-4^T^	99.8		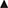
	H33-105	*Pelagibaca bermudensis* HTCC2601^T^	99.7		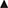
	H10-50	*Phenylobacterium falsum* AC-49^T^	98		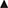
	H19-2	*Ponticaulis koreensis* DSM 19734^T^	98.2		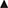
	H19-23	*Roseovarius mucosus* DSM 17069^T^	100	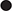	
	Rm01	*Ruegeria mobilis* NBRC 101030^T^	100	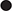	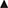
	H33-41	*Sinorhodobacter ferrireducens* SgZ-3^T^	100	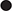	
	H18-57	*Sphingobium abikonense* NBRC 16143^T^	99.6	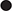	
	H18-18	*Sphingopyxis alaskensis* RB2256^T^	98.4		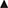
	H18-22	*Sphingopyxis italica* SC13E-S73^T^	99.9	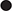	
	H10-48-1	*Thalassobaculum salexigens* DSM 19539^T^	99.7		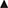
*Gammaproteobacteria*	H33-18	*Acinetobacter venetianus* RAG-1^T^	100		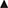
	H33-19	*Aestuariibacter aggregatus* WH169^T^	100		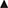
	H19-53	*Alcanivorax borkumensis* SK2^T^	99.5	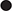	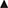
	H33-67	*Alcanivorax gelatiniphagus* MEBiC08158^T^	99.5		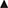
	H33-94	*Alcanivorax jadensis* T9^T^	98.7		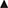
	H18-16	*Alcanivorax marinus* R8-12^T^	99.8		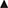
	H19-7	*Alcanivorax venustensis* ISO4^T^	100		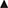
	H33-5	*Alteromonas macleodii* ATCC 27126^T^	99.8		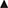
	H18-4	*Alteromonas marina* SW-47^T^	99.6		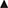
	H33-31	*Alteromonas tagae* BCRC 17571^T^	99.4		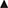
	H18-42	*Cobetia marina* DSM 4741^T^	100		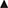
	H33-11	*Escherichia flexneri* ATCC 29903^T^	99.5		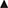
	H10-4	*Halomonas titanicae* BH1^T^	100	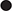	
	H33-82	*Klebsiella pneumoniae* ATCC 13884^T^	100		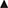
	H33-13-1	*Marinobacter adhaerens* HP15^T^	98.6		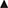
	H33-64	*Marinobacter algicola* DG893^T^	99.9		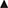
	H33-50	*Marinobacter goseongensis* En6^T^	99.8		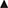
	H33-20	*Marinobacter hydrocarbonoclasticus* ATCC 49840^T^	99.7		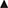
	H33-13-2	*Marinobacter koreensis* DD-M3^T^	98.9		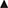
	H33-14-2	*Marinobacter litoralis* SW45^T^	96.8		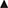
	H33-14-1	*Marinobacter maritimus* CK47^T^	97.6		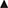
	H33-48	*Marinobacter sediminum* R65^T^	99.7		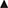
	H18-44	*Marinobacter similis* A3d10^T^	99.7		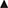
	H33-70-2	*Mediterranea mediterranea* DE^T^	98.7		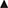
	H33-107	*Neptuniibacter caesariensis* MED92^T^	97.5		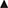
	H19-31	*Pseudoalteromonas hodoensis* H7^T^	100		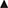
	H10-48-2	*Pseudoalteromonas shioyasakiensis* SE3^T^	97.9		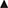
	H33-29	*Pseudoalteromonas spongiae* UST010723-006^T^	99.8		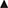
	H33-24	*Pseudomonas pachastrellae* KMM 330^T^	99.7		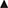
	H33-35	*Pseudomonas sabulinigri* J64^T^	98.6		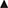
	H33-32	*Rheinheimera nanhaiensis* E407-8^T^	99.4		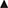
	H10-16	*Serratia arcescens* ATCC13880^T^	99.7	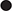	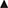
	H33-54	*Shewanella corallii* fav-2-10-05^T^	98.9		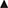
	H10-24	*Vibrio harveyi* ATCC 14126^T^	99.9	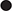	
	H18-1	*Vibrio neocaledonicus* NC470^T^	100	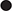	
	H10-21	*Vibrio sinaloensis* CAIM797^T^	99.1	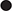	
	H10-39	*Vibrio variabilis* R-40492^T^	98.3	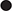	

*The identification of bacterial isolates was based on partial (∼800 bp) 16S rRNA gene sequences. The solid circle (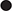) indicates AHL-producing bacterial isolate. The solid triangle (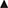) indicates AHL-degrading bacterial isolate*.

### AHL-Producing Profile of *R. mobilis* Rm01

**Figure 3:**
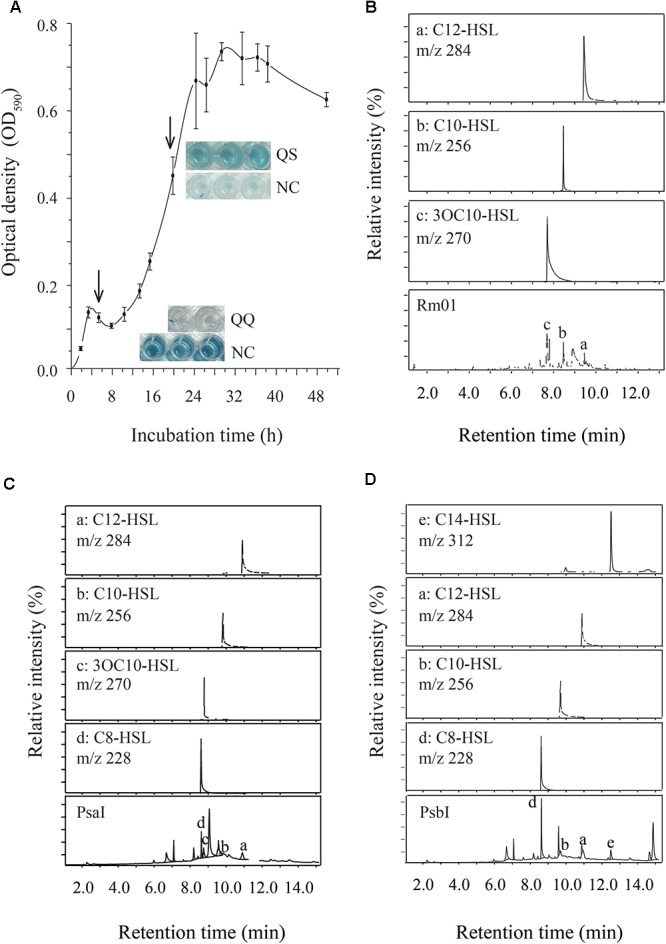
AHL-producing ability of *R. mobilis* Rm01. **(A)** The growth curve of Rm01. The arrows indicate the initiation time for QQ and QS activities. **(B–D)** GC-MS chromatograms in SIM mode at m/z 143 of cell-free supernatant extracts of wild-strain Rm01 and recombinant strains BL21 (DE3)/pET-24a–MG001458 (PsaI) and BL21 (DE3)/pET-24a–MG001460 (PsbI), respectively.

We applied biosensor strains and GC-MS analysis to detect the AHLs produced by Rm01. As indicated by the responses of the biosensor strains *C. violaceum* VIR24 (sensitive to AHLs with long acyl chains) and *C. violaceum* CV026 (sensitive to AHLs with short acyl chains), AHLs with long acyl chains were preliminarily inferred to be synthesized by Rm01 (Supplementary Figure 4). Following the observation of biosensor responses, the supernatant of Rm01 cultured for 24 h (Figure 3A) was extracted and analyzed by GC-MS. Three AHLs, 3OC10-HSL, C10-HSL and C12-HSL, were identified in Rm01 (Figure 3B), consistent with the results initially detected by the biosensor strains VIR24 and CV026.

In the genome of Rm01, two putative AHL synthases PsaI (Accession No. MG001458) and PsbI (Accession No. MG001460) (particle-associated symbiont locus A and B *luxI* homolog, respectively) were found and further verified as functional AHL synthases by heterogeneous expression in *E. coli* BL21 (DE3). PsbI shared 41% identity at the amino acid level with LuxI1 in *Dinoroseobacter shibae* DFL 12 (Neumann et al., 2013) while PsaI did not resemble to any reported AHL synthase. According to the BLASTP results, PsaI is conserved in *Rhodobacteracea* genomes, sharing at least 80% identities with the homologous proteins (Supplementary Figure 6). However, the homologs are automatically annotated as GNAT family N-acetyltransferase and no experimental evidence has been raised to verify their functions. The AHLs produced by PsaI and PsbI were also extracted and analyzed by GC-MS. As a result, *N*-octanoyl-L-homoserine lactone (C8-HSL), 3OC10-HSL, C10-HSL and C12-HSL were produced by PsaI (Figure 3C), while C8-HSL, C10-HSL, C12-HSL, and C14-HSL were produced by PsbI (Figure 3D). Except of C10-HSL, other AHLs identified in the recombinant strains were not detected in the AHL production background of the *E.coli* strain BL21 (DE3)/pET-24a (supplementary Figure 5). Most of the AHLs produced by PsaI and PsbI were included in the AHL-producing profile of Rm01. However, C8-HSL produced by both PsaI and PsbI, and C14-HSL produced by PsbI were not identified in the AHL extractions of Rm01, which might be explained by the differential acyl-ACP substrate pools between *R. mobilis* and *E. coli* (Zan et al., 2012).

### AHL-Degrading Ability of *R. mobilis* Rm01

**Figure 4:**
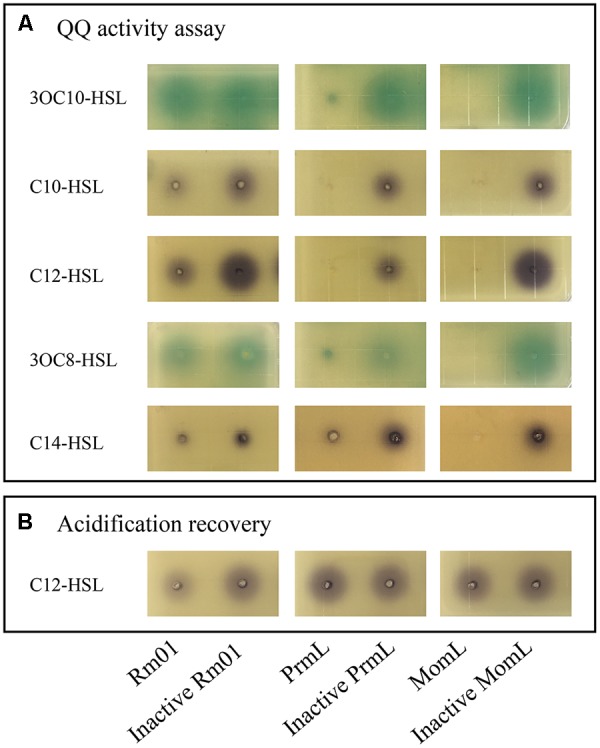
AHL-degrading ability of *R. mobilis* Rm01. The results are shown via well-diffusion assays supplemented with the AHL reporter strains A136 and VIR24. The degradation results for the self-generated AHLs 3OC10-HSL, C10-HSL and C12-HSL and non-self-generated AHLs 3OC8-HSL and C14-HSL are shown in frame **(A)**. Acidification recovery of hydrolyzed C12-HCL was conducted and is shown in frame **(B)**. Columns 1–6 represent the following samples, in order: Rm01 cell lysate; inactive Rm01 cell lysate; whole cells of BL21 (DE3)/pET-24a-MG001461 (PrmL); inactive whole cells of BL21 (DE3)/pET-24a (PrmL); AHL lactonase MomL (positive control); and inactive AHL lactonase MomL.

In addition to AHL-synthesizing ability, Rm01 also demonstrated AHL-degrading ability, termed as QQ. The QQ activity of Rm01 was detected at the beginning of the exponential growth phase indicated by both reporter strains A136 and VIR24 (Figures 3A, 4A). The AHL molecules C10-HSL, C12-HSL and C14-HSL were partially degraded, whereas 3OC8-HSL and 3OC10-HSL were not attenuated (Figure 4A). Based on these results, saturated AHLs were much more easily degraded by Rm01 than oxo-substituted AHLs.

One putative QQ lactonase gene, *prmL* (Accession No. MG001461) (particle-associated *R. mobilis* lactonase), was found in the genome of Rm01. PrmL expressed in heterologous system, degraded C10-HSL, C12-HSL and C14-HSL (Figure 4A) as well as 3OC8-HSL and 3OC10-HSL, which were not quenched by Rm01. Moreover, the enzymatic mechanisms of Rm01 and PrmL were revealed with an acidification experiment. Under acidic conditions, hydrolyzed C12-HSL by PrmL was completely recovered (Figure 4B), which indicated that PrmL was an AHL lactonase (Yates et al., 2002). The unrecovered C12-HSL might own to other unidentified QQ enzymes in Rm01 that can further hydrolyze ring-opened AHLs, e.g., AHL acylase.

### Exogenous AIs Regulate Biofilm Formation and Lipase Production in Rm01

**Figure 5:**
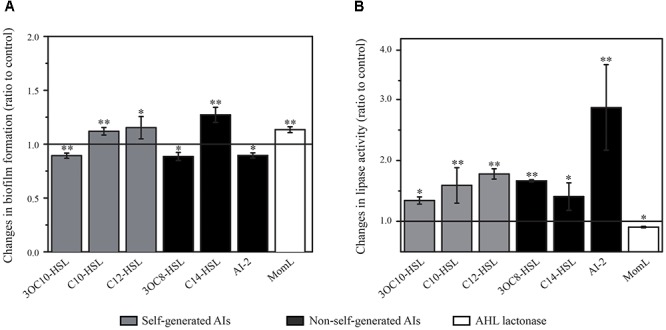
Changes in biofilm formation ability **(A)** and production of extracellular lipase **(B)** in *R. mobilis* Rm01 treated with self-generated 3OC10-HSL, C10-HSL and C12-HSL and non-self-generated 3OC8-HSL, C14-HSL, AI-2 and AHL lactonase MomL. The *y*-axis presents the ratios of experimental groups relative to control groups, and a value of 1.0 indicates no difference. The data are shown as the mean ± SD, and the difference between the amended groups and the control groups was calculated by Student’s *t*-test (^∗∗^*P* < 0.01; ^∗^*P* < 0.05).

Biofilm formation is essential for bacteria to colonize on marine particles and get access to resources (Dang and Lovell, 2016). To investigate the roles of different AIs play on biofilm formation in Rm01, we conducted feedback experiments using a variety of QS AI molecules (including self-generated AHLs and non-self-generated AHLs and AI-2). We demonstrated that the biofilm formation of Rm01 was reduced with 3OC10-HSL, 3OC8-HSL and AI-2, whereas increased with C10-HSL, C12-HSL, and C14-HSL (Figure 5A). Additionally, biofilm formation was also increased with an effective AHL lactonase MomL, which completely eliminated the intrinsic AHLs secreted by Rm01. The modest variations of biofilm mass may own to the counteraction by the intrinsic QS systems of Rm01. But according to the statistical analyses, the modulations of biofilm formation truly happened in Rm01 by sensing and responding to diverse AIs. The results might indicate bacterial adjustment during habitat colonization when encountering different bacterial species.

Bacterial EE production is important for the acquisition of nutrition, which is also the key factor regulating the marine particle degradation process. With amendment of above AIs, the growth of Rm01 was not affected whereas the lipase production in Rm01 was modulated. As is shown in Figure 5B, lipase activity in Rm01 was stimulated by all applied AHLs and AI-2, while inhibited by the AHL lactonase MomL. Above results indicated cooperations of Rm01 with other QS and QQ strains in nutrient acquisition, which ultimately affect the marine particle degradation process.

## Discussion

In this study, we extended our investigation of QS-regulated marine particle degradation by applying amendment experiments *in situ* and *in vitro*. Compared with previous findings (Hmelo et al., 2011; Krupke et al., 2016), the regulations of EE production in marine particles with exogenous AHLs were further observed on carbohydrases, xylosidase, galactosaminidase, β-glucosidase and mannosidase. RBG species are frequently observed on marine particles (Gram et al., 2002; Slightom and Buchan, 2009; Thiele et al., 2015; Table 1) and have been shown to possess AHL networks (Gram et al., 2002; Zan et al., 2014; Table 1), which indicates the significance of RBG species in QS-regulated metabolic mechanisms in niches. To obtain more concrete information, we identified the AHL-based QS network in an RBG species, *R. mobilis* Rm01, which possesses the additional QQ and AI-2 pathways. Further amendment experiments clearly demonstrated that biofilm formation and lipase production by Rm01 were tightly associated with its multiple QS networks described above. Our results here suggest that QS networks in Rm01 enable interspecies communications and coordinations of metabolisms in *R. mobilis*, which might be one of factors affecting marine particle degradation.

### QQ- and AI-2-Related Processes Are Important Components of the QS Regulatory Network of *R. mobilis* Rm01

*Ruegeria mobilis* Rm01 has a typical AHL-based QS system. In RBG species, many studies have demonstrated that QS systems participate in TDA production (Berger et al., 2011), flagellar motility and biofilm formation (Zan et al., 2012), which facilitate their high abundance (20%-40% of the 16S rRNA genes) in ocean waters (Moran et al., 2003; Buchan et al., 2005; Wagner-Dobler and Biebl, 2006). In addition, RBG species have been found more dominant in particle-associated fractions than in free-living fractions (Zan et al., 2014; Thiele et al., 2015). Though we did not measure the abundance of *R. mobilis* in this study, previous study has demonstrated that *R. mobilis*, one of the typical RBG species, was more abundant in particle-associated fractions than free-living fractions, with 40% and 6% occurrences respectively (Sonnenschein et al., 2016). However, the *R. mobilis* QS system has not been studied in detail. In this study, two AHL synthase genes, *psaI* and *psbI*, were detected in Rm01. According to genomic analysis, *psaI* is located downstream of a *luxR* transcriptional regulator *psaR* (Accession No. MG001459), whereas *psbI* is an “orphan” AHL synthase gene without a cognate transcriptional regulator. PsaI and PsbI fell into two new distinct clusters (Supplementary Figure 6) and exhibited few conserved residues with other identified AHL synthases (Supplementary Figure 7) according to neighbor-joining phylogenetic tree and multiple-sequence alignment analyses, respectively. We propose that the QS system in Rm01 represents a new, previously undiscovered QS system in RBG species.

QQ might be another important process for the adaptation of Rm01 to marine particles. Previous studies demonstrated that many strains capable of AHL interference have been isolated from sediments, biofilms, the surface of the alga *Fucus vesiculosus* (Romero et al., 2012) and the Mediterranean seagrass angiosperm Posidonia oceanica (Blanchet et al., 2017), and marine particles (Table 1) in which QS activity is easily concentrated. The QQ processes in dense microbial niches limit the coordination of antagonistic bacteria and even affect vital activities of their phytoplankton host (Tait et al., 2009; Rolland et al., 2016). Coordinated mechanism achieved by QS and QQ systems is essential for bacterial survival. QS systems in *Burkholderia pseudomallei* (Lumjiaktase et al., 2006), *Vibrio cholera* (Joelsson et al., 2007) and *Pseudomonas aeruginosa* (García-Contreras et al., 2015) could enhance their stress tolerance and even affect bacterial ecology by promoting their resistance to QQ strains (García-Contreras et al., 2015). In the extremophilic bacterium *Deinococcus radiodurans*, the QS and QQ systems cooperate in regulating gene to resist oxidative stress (Lin et al., 2016). Combined QS and QQ systems have not been reported in RBG species yet. Our work on the cooperation of QS and QQ processes in Rm01 extends the existing evidence regarding successful regulatory mechanisms in marine particles.

An AI-2-based QS network enables cross-species communications on a wider scale. AI-2 synthase LuxS homologs have been found in 537 out of the 1402 sequenced bacterial genomes (Pereira et al., 2013). However, in Rm01 or even other RBG species, neither the signal molecule AI-2 (Supplementary Figure 8) nor its synthase LuxS homolog has been identified. In our experiments, lipase production and biofilm formation in Rm01 were regulated by AI-2 (Figure 4), which suggested the existence of AI-2 perception pathway. Rm01 genome annotation results conducted on the RAST server (Aziz et al., 2008) identified homologs of periplasmic AI-2 binding protein LsrB (Accession No. MG00148), proteins involved in an AI-2 internalization system (LsrA, Accession No. MG00145; LsrC, Accession No. MG00146; LsrD, Accession No. MG00147), the AI-2 phosphokinase LsrK (Accession No. MG001463) and the *lsr* repressor LsrR (Accession No. MG001464), which illustrates the genetic capabilities of Rm01 to respond to exogenously supplied AI-2. Coincidently, a similar pathway was observed in a plant symbiont, *Sinorhizobium meliloti*, which is unable to produce but nonetheless responds to AI-2 in the surrounding environment (Pereira et al., 2008). By “eavesdropping” on AI-2 produced by other species, the strain was capable of interfering with AI-2-regulated behaviors such as virulence. Although AI-2 has not been detected in marine particles, it is very likely to occur due to the prevalence of AI-2-producing *Vibrio* species (Bassler et al., 1994) in marine particles (Hmelo et al., 2011; Jatt et al., 2015; Table 1). The AI-2 pathway might facilitate Rm01 competing against antagonistic species in the microflora. Until now, research on AI-2-regulated mechanisms in marine bacteria has been rare, with the exception of studies investigating *Vibrio* species. Thus, our investigations of AI-2-regulated mechanisms in Rm01 might provide more hints that reveal the roles of AI-2 systems in marine bacteria and interspecies communications in marine particles.

### QS-Regulated Biofilm Formation and EE Production Promote the Adaption of Rm01 to Marine Particles

Biofilms are essential for heterotrophic bacteria colonizing marine particles, facilitating their access to resources (Dang and Lovell, 2016) and maintaining the integrity and activity of secreted bacterial EEs (Flemming and Wingender, 2010). Moreover, the enrichment of bacteria in a biofilm stimulates bacterial QS, which in turn affects biofilm formation and EE production in microflora (Dang and Lovell, 2016). Bacterial biofilm formation, EE production and bacterial QS are undoubtedly highly interactive and inseparable in marine particles.

Previous studies demonstrated that biofilm formation is characteristic feature of RBG species in transition between motile and sessile life stages, which is regulated by a second messenger c-di-GMP (Hengge, 2009; Mcdougald et al., 2011; D’Alvise et al., 2014). Here, we suggest that the transition between motile and sessile states in *R. mobilis* Rm01 is also regulated with self-generated and non-self-generated AIs by modifying biofilm formation. Notably, biofilm formation was inhibited by self-generated AHL 3OC10-HSL, which might accumulate faster than C10-HSL and C12-HSL in early proliferation period due to its QQ activities. Reduced biofilm mass enables Rm01 searching for a substrate or a host more effectively. Once settle down, self-generated C10-HSL and C12-HSL accumulated faster and subsequently up-regulated the biofilm formation in Rm01, facilitating its persistence in high organic substrate (e.g., marine particles).

Additionally, the transition between sessile and motile life of Rm01 is also affected by non-self-generated AIs produced by bacteria in marine particles. Bacteria secreting 3OC8-HSL and AI-2 (e.g., *Vibrio* species) (Bassler et al., 1997; Surette et al., 1999; Rajput et al., 2016) might resist the colonization of Rm01 by inhibiting its biofilm formation, while C14-HSL synthesizing species (e.g., RBG species) (Ziesche et al., 2015) might assist the colonization of Rm01 by promoting its biofilm formation. Relying on the eavesdropping behaviors, the strain Rm01 is expected to colonize marine particles more selectively by developing a biofilm matrix with commensal rather than antagonistic bacteria.

All tested AIs in this study increased lipase production in Rm01. We suggest that bacteria in marine particles secreting AIs could impact lipase production in Rm01. Thus they may together affect marine particle degradation process by QS systems. In this study, we also found that bacterial lipase was prominent in tested EEs from marine particles as well as the bacterial isolates (Supplementary Table S2; Martinez et al., 1996). Lipids in marine environment are derived from cell membranes of dead organisms and a variety of other biological sources (Martinez et al., 1996), which lets them to be one of the inevitable components in marine particles (Lee et al., 2004). The hydrolyzed products of lipids, glycerol and fatty acids, are readily usable sources of energy for heterotrophic microbial communities (Zoppini et al., 2005). Therefore, the QS-regulated production of bacterial lipase is clearly meaningful to bacterial survival in dense microbial habitats and further affects the cycling of organic matter in the oceans.

### Future Perspectives

In this study, *R. mobilis* Rm01 was proposed as the model strain to reveal the mechanisms underlying QS-regulated degradation processes of marine particles. Rm01 was capable of perceiving and interfering with diverse exogenous AIs to regulate its biofilm formation and lipase production. Our results demonstrate the molecular basis how Rm01 intercommunicate with other species and affects the marine particle degradation process to a certain extent. We suggest that *R. mobilis* play significant roles in marine particle degradation through bacterial interactions realized by QS networks. However, details of the pathways need to be better probed. In future investigations, transcriptome analysis is required to reveal the differential gene expression in detail. Metagenomic and metatranscriptomic analyses are also expected to characterize microbial structure and unravel QS pathways in marine particle microflora in avoid of culturability bias.

## Author Contributions

X-HZ, YS, and KT contributed to the conception and design of the study. YS and YZ performed the molecular biological and biochemical experiments. YS, JL, and YW performed the statistical analysis. YS and YZ wrote the first draft of the manuscript. All authors contributed to manuscript revision, read and approved the submitted version.

## Conflict of Interest Statement

The authors declare that the research was conducted in the absence of any commercial or financial relationships that could be construed as a potential conflict of interest.
