# Supplementary material for: Quorum Sensing System of Ruegeria mobilis Rm01 Controls Lipase and Biofilm Formation
Source: Front Microbiol. 2019 Jan 9;9:3304. doi: 10.3389/fmicb.2018.03304 (PMC6333666; doi:10.3389/fmicb.2018.03304)
Supplement: Supplementary file 1 [file Data_Sheet_1.PDF]

Quorum sensing system of *Ruegeria mobilis* Rm01 controls lipase and biofilm formation.

Ying Su[1,3], Kaihao Tang[1], Jiwen Liu[1, 2], Yan Wang[1,2], Yanfen Zheng[1] and Xiao-Hua Zhang[1, 2*]

[1]College of Marine Life Science, Ocean University of China, Qingdao 266003, China

[2]Laboratory for Marine Ecology and Environmental Science, Qingdao National Laboratory for Marine Science and Technology, Qingdao 266071, China

[3]Weifang Engineering Vocational College, Weifang 262500, China

***Author for correspondence:**

Xiao-Hua Zhang, College of Marine Life Science, Ocean University of China, 5 Yushan Road, Qingdao 266003, China; Tel/Fax: +86-532-82032767; Email: xhzhang@ouc.edu.cn

Running title: Quorum sensing regulates marine particle degradation

Supplementary Table 1: Primers used in this study.

Primer name	Restriciton Enzyme	Sequence (5'-3')
prmaI F	BamHI	CGGGATCCATGCACGATGGCGCGCCACAGTT
prmaI R	XhoI	CCCTCGAGTCCGGTGGTGGTTCCACG
prmbI F	BamHI	CGGGATCCATGCAGTCAACTGAAATCACT
prmbI R	HindIII	CCAAGCTTGCCGGCGTTGCGAATGC
prml F	SacI	CGAGCTCATGACCCTTTCCCGTAGAACTCTTT
prml R	Not I	ATTTGCGGCCGCAAGCCCGAACTGCCATGGTG

Engineered restriction sequences are underlined

Supplementary Table 2: Activity ranges of hydrolytic enzymes in marine particles and proportions of isolates expressing enzyme activities

Enzyme	Activity range (nM·h^{-1}·L^{-1})	% of Isolates expressing activity	*n*
α-glucosidase	2.0-171	35.4	96
β-glucosidase	30-221	35.4	96
β-xylosidase	0.0-88	5.6	36
mannosidase	0.0-67	2.8	36
cellulase	2.0-61	13.9	36
galatosaminidase	4.0-37	8.3	36
chitobiase	7.0-184	34.4	96
aminopeptidase	379-1634	59.4	96
lipase	982-11,193	100.0	96
phosphatase	452-3230	88.5	96

Letter *n* indicates the quantity of the tested bacterial isolates.

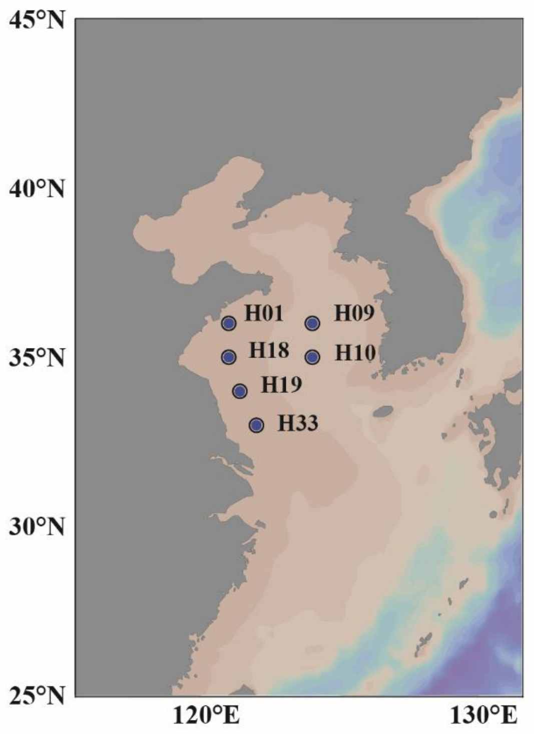

Supplementary Figure 1: Stations for marine particle collection. The circles represent the sample collection sites.

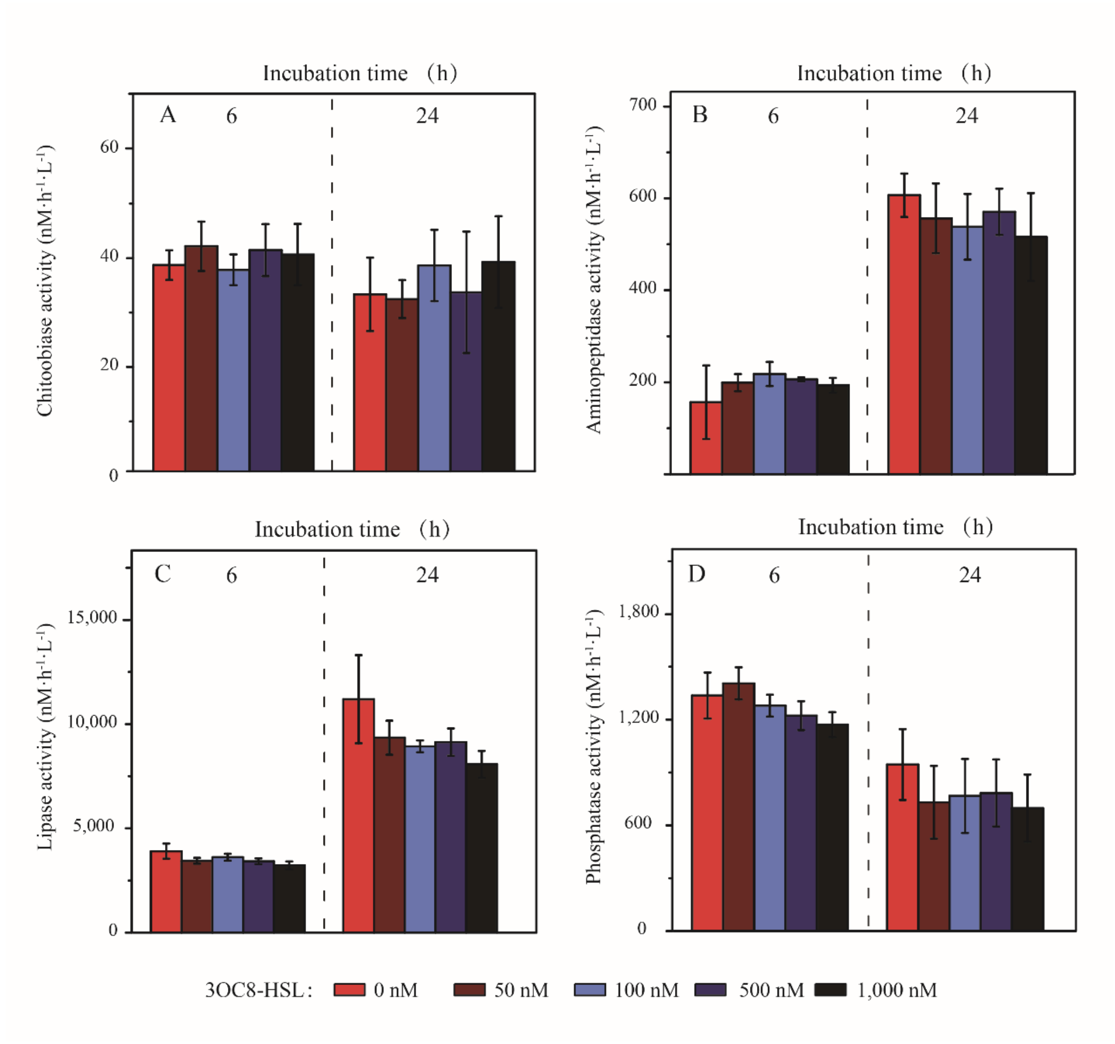

Supplementary Figure 2: *In situ* chitobiase (A), aminopeptidase (B), lipase (C) and phosphatase (D) activities in marine particles treated with 3OC8-HSL (the final concentrations of 3OC8-HSL are shown in different colors). The data are shown as the means ± standard deviation (SD). No difference was observed between the 3OC8-HSL-treated groups and the untreated control groups by Student's *t*-test calculations.

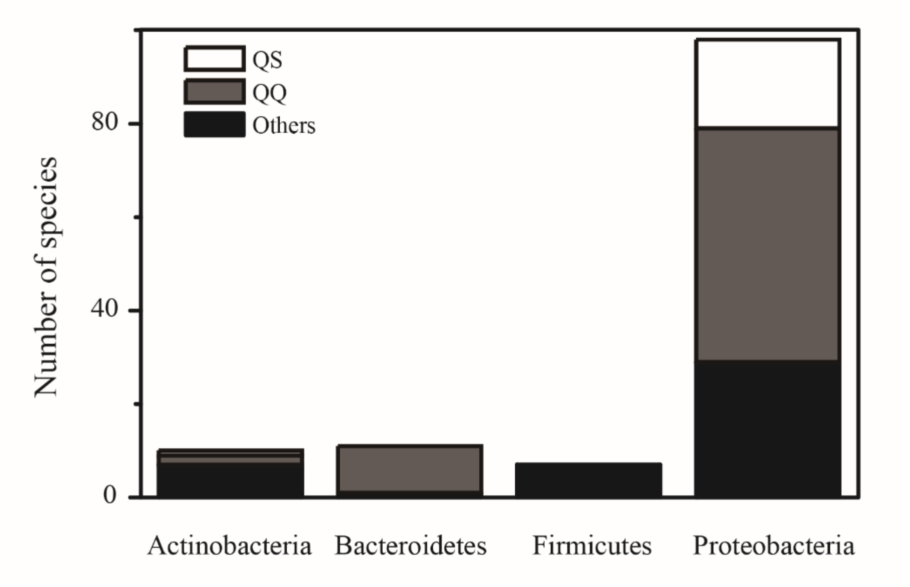

Supplementary Figure 3: Cultivable bacterial community composition in marine particles, shown at the phylum level. In each phylum, proportions of species possessing quorum sensing (QS) and quorum quenching (QQ) abilities are indicated in different colors.

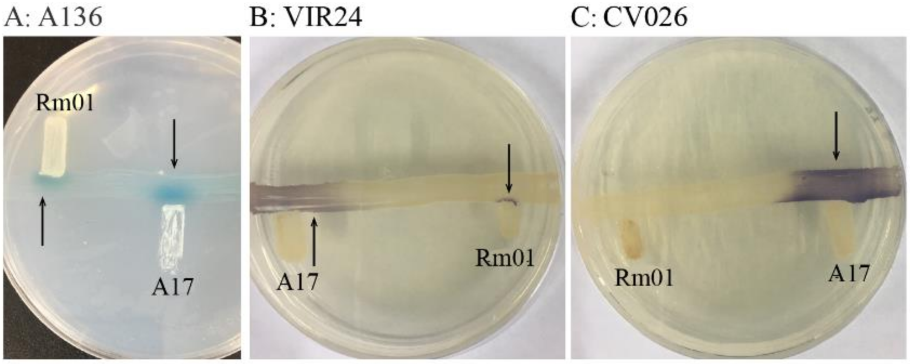

Supplementary Figure 4: Detection of AHL-producing ability by the reporter strain *Agrobacterium tumefaciens* (pCF218) (pCF372) A136 (A), *Chromobacterium violaceum* VIR24 (B) and *C. violaceum* CV026 (C). Abbreviations: Rm01, strain *Ruegeria mobilis*; A17, strain *Aeromonas salmonicida* (positive control). Both A136 and VIR24, except of CV026, positively reacted to AHLs produced by Rm01, which indicated that only long acyl-chain AHLs were produced by Rm01.

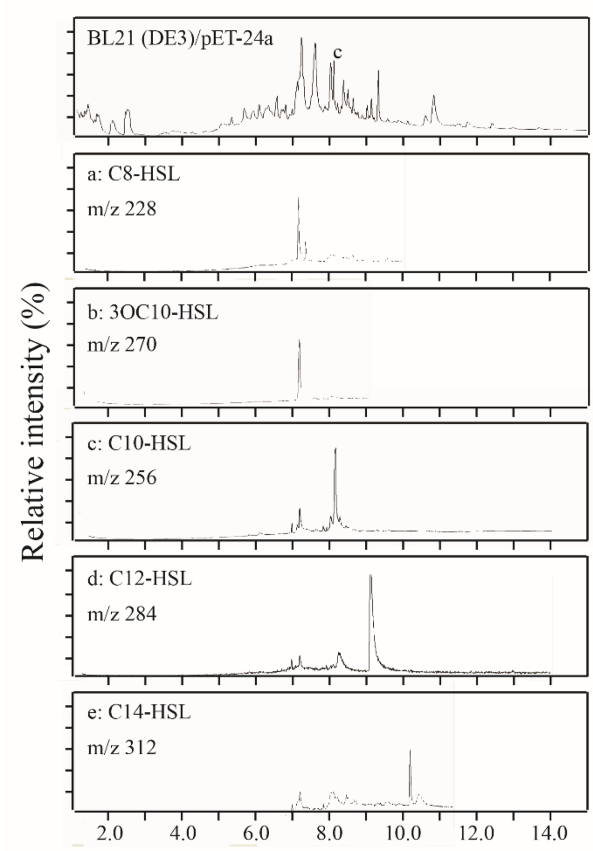

Supplementary Figure 5: GC-MS chromatograms in SIM mode at m/z 143 of cell-free supernatant extracts of the *Escherichia coli* BL21 (DE3)/pET-24a.

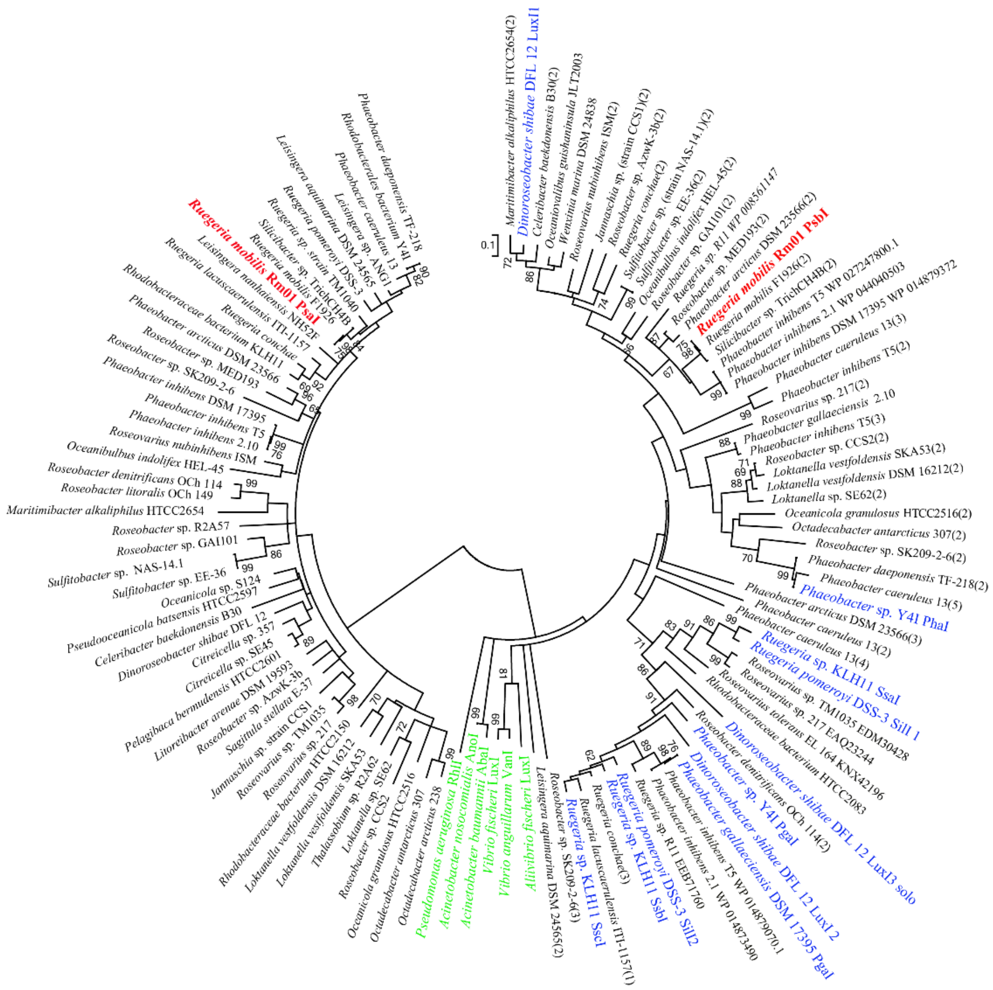

Supplementary Figure 6: Phylogenetic tree of AHL synthases PsaI, PsbI (shown in red color) and their respective BLAST hits employing neighbor-joining method with the MUSCLE program in the MEGA software package (1,000 bootstrap replicates). Bootstrap coefficients below 60% were not shown. Scale bar, 0.1 substitutions per amino acid position. The items marked in blue and green colors are reported AHL synthases from the RBG and other distinct clades.

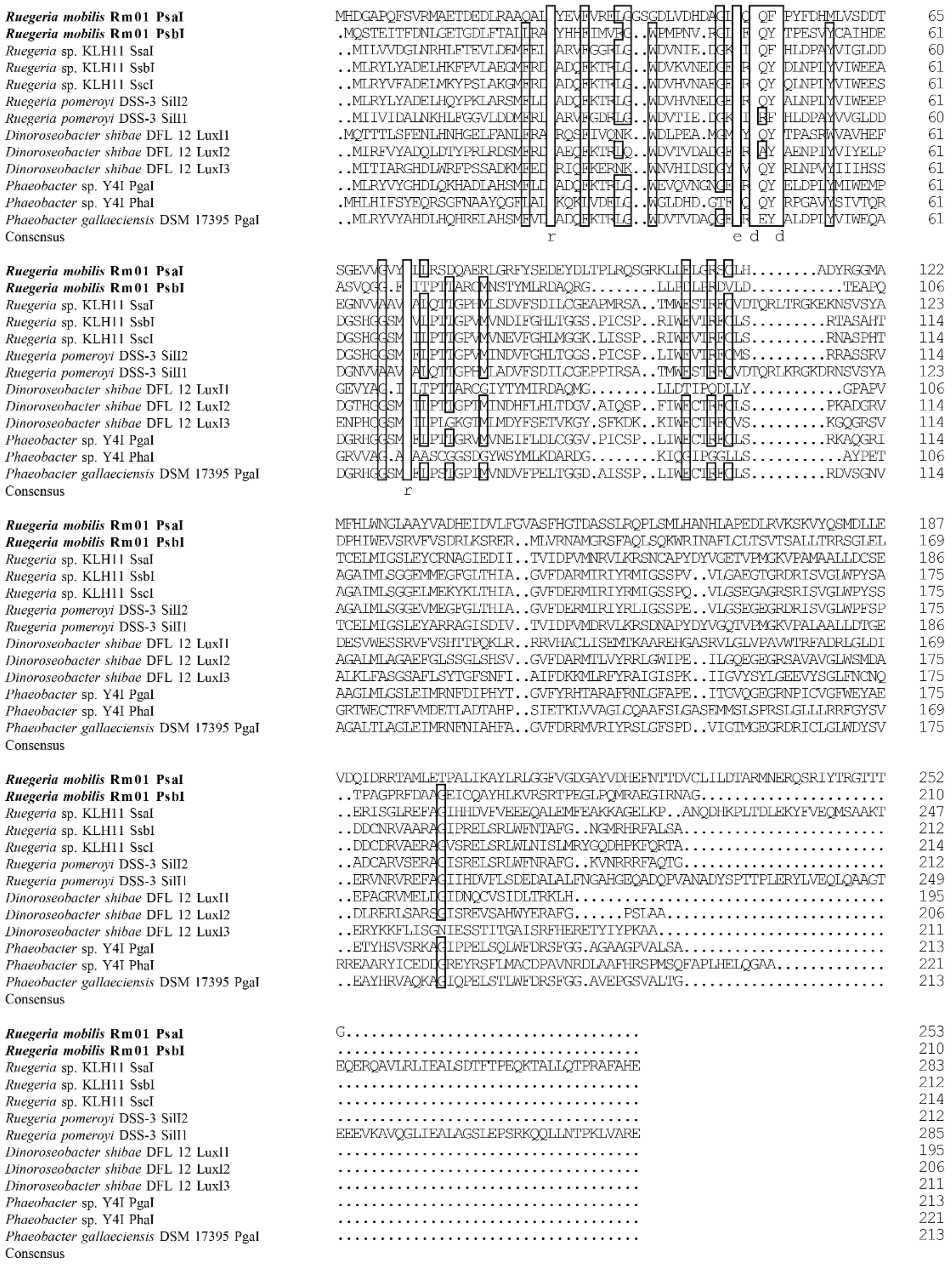

Supplementary Figure 7: Multiple-sequence alignment of amino acid sequences of PsaI and PsbI and the identified AHL synthases in the RBG. Sequence alignment was performed by the MUSCLE program in the MEGA software package. Empty rectangles indicate the conserved residues in some AHL synthases.

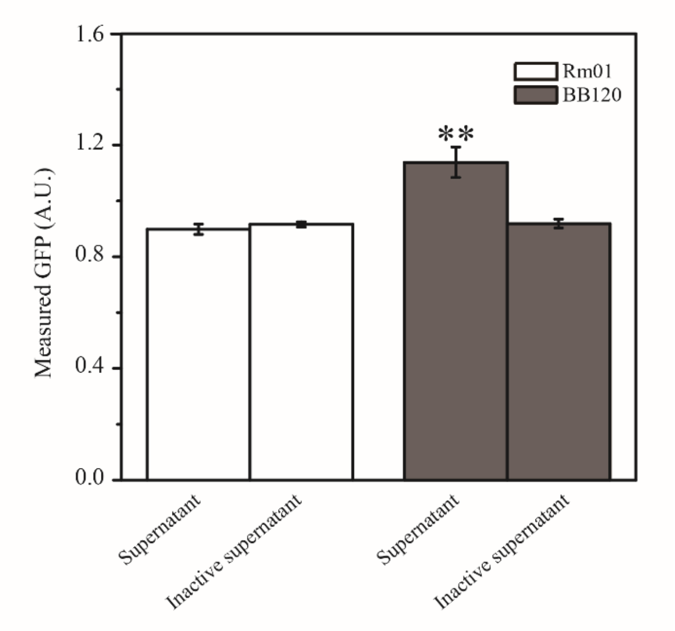

Supplementary Figure 8: Detection of AI-2-producing ability by the reporter strain *Vibrio harveyi* LuxPQ$^+$ (TL88). The experiments were conducted as described previously (Long *et al.*, 2009) with a few modifications. In brief, a cell-free supernatant of Rm01 and BB120 (wild-type strain of *V. harveyi*) was mixed with the reporter strain TL88 and maintained at 28 °C for 12 h. After co-incubation, the GFP fluorescence intensity of TL88 was measured with a Fluoroskan Ascent FL multi-well plate reader (Thermo) with excitation and emission wavelength at 485 nm and 538 nm respectively. Boiled supernatant served as the negative control. BB120 served as the positive control. A.U. indicates arbitrary units.

References

Long, T., Tu, K.C., Wang, Y., Mehta, P., Ong, N.P., Bassler, B.L., and Wingreen, N.S. (2009) Quantifying the integration of quorum-sensing signals with single-cell resolution. *PLoS Biol* **7**: 640-649.
